# Early and persistent high level of PS 100β is associated with increased poor neurological outcome in patients with SAH: is there a PS 100β threshold for SAH prognosis?

**DOI:** 10.1186/s13054-016-1200-1

**Published:** 2016-02-03

**Authors:** Hervé Quintard, Sébastien Leduc, Patricia Ferrari, Isabelle Petit, Carole Ichai

**Affiliations:** 1 Intensive Care Unit, Hôpital Pasteur 2, Nice University Hospital, 30 Voie Romaine, Nice, 06000 France; 2 Department of Biochemistry, Hôpital Pasteur 1, Nice University Hospital, Nice, 06000 France; 3 IRCAN, UMR INSERM U10891-CNRS 7284, Faculté de Médecine, Nice Sophia-Antipolis University, Nice University Hospital, Nice, 06000 France

**Keywords:** Surrogate marker, Aneurysmal subarachnoid hemorrhage, S100 protein, Neuron specific enolase, Outcome

## Abstract

**Background:**

Protein S100β (PS100 β) and neuron specific enolase (NSE) have been described as biological markers of neuronal damage. The purpose of our study was to assess the prognosis thresholds of these biomarkers in subarachnoid aneurysmal hemorrhage (SAH).

**Methods:**

Forty eight patients admitted following SAH were treated by endovascular coiling. Initial neurologic severity was assessed using the World Federation Neurologic Surgeons (WFNS), Fisher grades, initial Glasgow coma scale (GCS) and SAPS II. PS100β and NSE plasma concentration were measured daily within the first week. The primary endpoint of the study was the 6-month Glasgow Outcome Score (GOS) dichotomized as poor (GOS 1–3) or good (GOS 4–5).

**Results:**

A poor outcome at 6-months was associated with significant higher levels of S100β value from day 1 to day 7, whereas NSE values were significantly higher from day 5 to day 7. Best threshold value, for prognosis, was obtained at day 5 for PS100β >0.13 μg/L (specificity 0.95 95 % confidence interval (CI) 0.74–1; sensitivity 0.83 95 % CI 0.65–0.93) and day 7 for NSE >14.5 μg/L (specificity 0.90 95 % CI 0.67–0.98); sensitivity (0.69 95 % CI 0.51–0.83)). After multivariate logistic analysis, only PS100β at day 5 and SAPS II enabled to predict neurological outcome at 6 months (*p* <0.05).

**Conclusion:**

PS100β >0.13 μg/L at day 5 is an independent predicting factor of poor neurological outcome at 6 months following SAH. This result could support the use of this biomarker at the acute phase of SAH to help physician determine the prognosis.

**Electronic supplementary material:**

The online version of this article (doi:10.1186/s13054-016-1200-1) contains supplementary material, which is available to authorized users.

## Background

Subarachnoid aneurysmal hemorrhage (SAH) has a 30-day mortality rate of 45 % with approximately half of the survivors sustaining irreversible brain damage [1]. It is a real public health problem and early identification and quantification of cerebral damage after injury can be challenging. Initial clinical status developed by the World Federation of Neurosurgical Society (WFNS) [2] has been associated with prognosis [3], but physical examination is not sufficient to assess and accurately monitor clinical status in comatose patients or those under general anesthesia. The initial imaging Fisher score or modified Fisher scale [4] is usually obtained in clinical practice. This classification has been proposed to assess the risk of vasospasm after SAH but not direct assessment of prognosis.

Protein S100β (S100β) is a 21 kDa protein with a biological half-life of 2.5 h [5]. Neuron specific enolase (NSE) is an isoenzyme found in mature neuron. PS100 β or NSE were important innovations in the field of neurologic prognosis in the past decade. These biomarkers have been described to increase after stroke [6] or traumatic brain injury [7] and are correlated with neurologic damage and prognosis [8]. Several trials have reported that they are also good markers of delayed prognosis in SAH [9, 10]. Indeed, mean and peak values of serum S100β provide the ability to distinguish patients with good and those with poor outcome within 8 days after SAH [11–13]. NSE is also acknowledged as a good predictor of late prognosis in this setting [10]. However, the most appropriate timing for monitoring these biomarkers is not clearly defined. Stranjalis et al. measured daily S100β and concluded that the first initial measurement is correlated with neurological outcome in patients with SAH [14]. In another study, Oertel et al. proposed 3-day screening of S100β to predict outcome or vasospasm [10]. Others studies report that high mean 8-day S100β values predict poor outcome after 1 year [9, 15].

Thus, the purpose of this study was to investigate daily serum S100β and NSE measurements over 7 days after SAH in order to assess the most accurate timing allowing determination of neurological prognosis.

## Methods

This prospective observational study was approved by our local ethics committee (Comité de Protection des Personnes, Sud Méditérranée V, 2011-A00231-40). In accordance with the Helsinki declaration, written informed consent was obtained from the patients or the patient’s next of kin.

### Ethical approval

All procedures performed in studies involving human participants were in accordance with the ethical standards of the institutional research committee and with the 1964 Helsinki declaration and its later amendments or comparable ethical standard. Informed consent was obtained from all individual participants.

### Patients

The inclusion period was from July 2011 to July 2012. Inclusion criteria were: 1) a recent clinical history of SAH before admission (within a maximum of 48 h following the first symptoms) with evidence of bleeding on computed tomography (CT) and presence of an aneurysm on cerebral angiography; 2) age ≥18 years; and 3) treatment by coiling within 72 h after the beginning of symptoms. In our institution, coiling is systematically performed in SAH patients as the primary treatment and surgical clipping is intended as a second-line treatment, or for special indications such as anatomical considerations avoiding coiling or intraparenchymatous hematoma requiring surgical treatment. All patients were admitted to an Intensive Care Unit after initial treatment. Exclusion criteria were admission later than 48 h after bleeding, traumatic SAH, coiling later than 72 h after admission and therapeutic abstention.

### Clinical and CT evaluation

We conducted a double-blind observational study i.e., the physicians and nurses in charge of patients and the outcome assessors were blinded to biomarker levels. At admission, clinical neurological severity was assessed using the WFNS score [2] as follows: 1 = Glasgow coma scale (GCS) of 15; 2 = GCS score of 13–14 and no motor deficit; 3 = GCS score 13–14 and any motor deficit or aphasia; 4 = GCS 7–12, with or without motor deficit; and 5 = GCS 3–6 with or without motor deficit. The initial CT scan was reviewed by an independent radiologist blinded to clinical history, therapy and biomarker values. He classified patients using the modified Fisher score [4] as follows: grade 1 = focal or diffuse thin SAH, no intraventricular hemorrhage (IVH); 2 = focal or diffuse thin SAH with IVH; 3 = thick SAH present, no IVH; grade 4 = thick SAH present with IVH; and grade 5 = IVH or intracerebral hematoma with clot. Neurologic outcome was assessed using the Glasgow outcome scale (GOS) score at 6 months assessed by phone interview by an anesthesiologist, blinded to the biomarker results. The GOS was defined as follows: 1 = death; 2 = persistent vegetative state; 3 = severe disability; 4 = moderate disability; 5 = good recovery [4] (Additional file 1).

### Clinical management

All patients included in the study were treated by the coiling technique. They were managed according to the international guidelines. Briefly, all patients received intravenous nimodipine at a dose of 2 mg/h, then switched to oral nimodipine as soon as possible (200 mg × 6/d). They were monitored by an arterial and central venous catheter to target a normovolemic state, systolic arterial pressure above 130–140 mmHg, normoglycemia and normothermia. Carbon dioxide partial pressure (PaCO_2_) was maintained between 35–40 mmHg and SpO_2_ above 97 %. An external ventricular drain (Integra Life Science® Saint Priest, France) was inserted in the case of hydrocephalus being observed on CT. The line was connected to an external strain gauge allowing continuous monitoring of intracranial pressure (ICP). Episodes of raised ICP were treated by cerebrospinal fluid drainage as a first line of treatment, ventilation control to maintain normocapnia, normoxia (see supra), and as a second line of treatment with deeper sedation. CT was performed whenever clinical deterioration occurred, to search for secondary complications such as hydrocephalus or ischemia, and systematically at day 7 after admission. Delayed cerebral ischemia (DCI) was defined as cerebral infarction identified on CT or magnetic resonance imaging (MRI), after exclusion of procedure-related infarctions.

Vasospasm was suspected if there was clinical deterioration, fever, appearance of new symptoms, mean transcranial Doppler (Vivid S5, General Electric®) velocities above 120 cm/s or a daily change in mean transcranial Doppler velocities above 50 cm/s, and confirmed by cerebral angiography. Angioplasty was used as second-line therapy when nimodipine was judged insufficient. In our institution, no clinical care management was based on biomarker level.

### Data collected

During the 7 days of monitoring, hemodynamic measurements (arterial pressure and central venous pressure), urinary output, oxygen saturation (SpO_2_), and expiratory carbon dioxide (FeCO_2_) were continuously monitored (Philips Intellivue system®). The simplified acute physiology score (SAPS II) was collected during the first 24 h post SAH.

### NSE and S100B protein measurements

Arterial samples were collected daily for protein S100β (PS100 β) and NSE measurements within 7 successive days after ICU admission. An aliquot of the arterial blood sampled for the usual measurements was centrifugated in the central laboratory and frozen for further analysis. PS100β and NSE were determined in the serum with an electrochemiluminescence immunoassay kit using a sandwich technique (Liaison, DiaSorin®, S.p.A, Sallugia, Italy); within-assay and between-assay coefficients of variation were 3.1 % and 8.2 %, respectively for PS100β and 0.9 %, and 5.3 % for NSE. Sensitivity for PS100 β was 0.02 μg/L and 0.04 μg/L for NSE. Reference values were <0.15 μg/L for PS100 and <18.3 μg/L for NSE.

### Statistical analysis

Only patients who completed the total follow up of the study (7 days for biomarker measurements and 6 months for neurological outcome) were included in the statistical analysis. For statistical purposes, the WFNS, Fisher and GOS scores were dichotomized into those with slight–moderate severity (WFNS score 1–2 and Fisher score 1–3) vs profound severity (WFNS score 3–5, Fisher score 4–5), and those with good outcome (GOS 4–5) vs those with poor outcome (GOS 1–3). Data were expressed as mean ± standard deviation (SD), median ± interquartile range (IQR) for continuous variables and percentages for categorical variables. Comparisons were performed using the unpaired Student *t* test for normal variation variables, the Mann Whitney test for non-parametric variables, and the Chi^2^ test for categorical variables. A *p* value <0.05 was considered significant. One-way analysis of variance (ANOVA) for repeated measurement was performed followed by Tukey’s post-hoc test to integrate covariates, as the WFNS score and SAPS II had been obtained for the 7 days of monitoring (time effect, group effect, interaction effect). Cutoffs of PS100β and NSE values for each 7 days of monitoring were obtained by receiver operating characteristic (ROC) curve analysis according to prognosis at 6 months. We computed specificity, sensitivity, predictive positive and negative value and accuracy, with the 95 % confidence intervals, for each day. For univariate analysis for the evaluation criterion, the 6-month outcome as assessed using the GOS score, was performed by the creation of contingency tables and Student’s test for the following variables: initial WFNS, best cutoff for PS100β and NSE, SAPS II. The SAPS II and WFNS score have been already described as predictive for neurological outcome [16, 17]. Multivariate analysis was performed using backward stepwise logistic regression to enter variables that yielded *p* values <0.1 in the univariate analysis, to identify factors that independently predicted the 6-month outcome [18]. Statistical analysis was performed using XLSTAT version 2013.2.01 (Addinsoft®, New York, NY, USA), Stat View software®.

## Results

### Characteristics of patients and comparison according to severity at 6 months

A total of 58 patients were consecutively enrolled in the study. Among them 10 patients were excluded from analysis because they died during the first week. A total of 48 patients completed the follow-up study period and were analyzed. None of them underwent a surgical procedure. Characteristics of patients, according to 6-month outcome are summarized in Table 1. The initial GCS was 14 [3–14, 19]. According to the WFNS score 28 patients had slight–moderate severity and 20 profound severity at admission (WFNS score 1, 33 %; score 2, 27 %; score 3, 8 %; score 4, 13 % and score 5, 19 %). The Fisher score was mainly 4 (75 %). The main aneurism location was on the anterior communicant artery (54 %). Hydrocephalus was present in 66 % of patients and needed external derivation.

**Table 1:** Demographic and characteristics of patients according to 6-month outcome

	GOS 1–3	GOS 4–5	*p*
	n = 19	n = 29	
Age, years	55 ± 12	50 ± 10	0.14
Gender			
Women	11	19	0.6
Men	8	10	
SAPS II ± IQR	34 ± 30	20 ± 13	<0.01
GCS ± IQR	14 ± 6	14 ± 2	0.01
Initial WFNS score, n (%)			0.02
1–2	6 (32)	22 (75)	
3–5	13 (68)	7 (25)	
Initial Fisher score, n (%)			0.8
1–2	2 (11)	4 (14)	
3–5	17 (89)	25 (86)	
Aneurism location, n (%)			0.1
ACA + AComA	13 (68)	17 (58)	
CA + PComA	1 (5)	8 (28)	
MCA	2 (11)	4 (14)	
Vertebrobasilar system	3 (16)	0	
Intraventricular hemorrhage, n (%)	15 (79)	17 (60)	0.2
Intraparenchymentous hematoma, n (%)	8 (42)	6 (20)	0.1
Hydrocephalus, n (%)	15 (79)	17 (60)	0.2
Initial neurogenic lung edema, n (%)	1 (5)	3 (10)	0.4
Delayed cerebral ischemia, n (%)	13 (68)	3 (11)	<0.001
Length of stay in ICU Median (range)	37 (8–136)	15(3–68)	0.1
Hospital mortality, n (%)	9 (47)	0	<0.001

Outcome: Glasgow outcome scale (*GOS*), where a score of 1 indicates death. *SAPS* simplified acute physiology score, *GCS* Glasgow coma score, *WFNS* World Federation of Neurosurgical Society, ACA cerebral anterior artery, *AComA* anterior communicant artery, *CA* carotid artery, *PComA* posterior communicant artery, *MCA* middle cerebral artery

Among the patients 27 % presented with angiographic vasospasm during the study period, and 54 % with delayed cerebral ischemia. The global mortality rate was 14.5 % in the ICU and 18.5 % during the hospital stay. At 6 months 29 patients (60 %) had a good recovery (15 patients with a GOS of 4 and 14 patients with a GOS of 5) and 19 (40 %) a poor outcome (10 GOS 3 and 9 GOS 1). We investigated the occurrence of DCI occurring during SAH according to prognosis group. We observed a rate of DCI in the poor prognosis group of 68 % compared to 10 % (*p* <0.05).

### Time course values of S100β and NSE within the first week following SAH in good and poor outcome groups

**Fig. 1:**
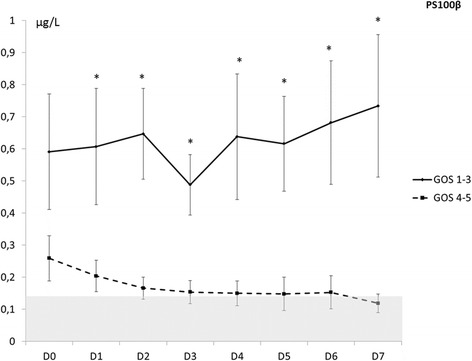
Time course values of S100β within the first week after subarachnoid hemorrhage in groups with good or poor outcome (*p <0.05). *GOS* Glasgow outcome scale

At day 1 after SAH, PS100β was significantly higher in the poor recovery group compared to patients with a good prognosis (0.6 vs 0.2 μg/L; *p* = 0,01). This significant difference persisted within the first week of monitoring (Fig. 1).

**Fig. 2:**
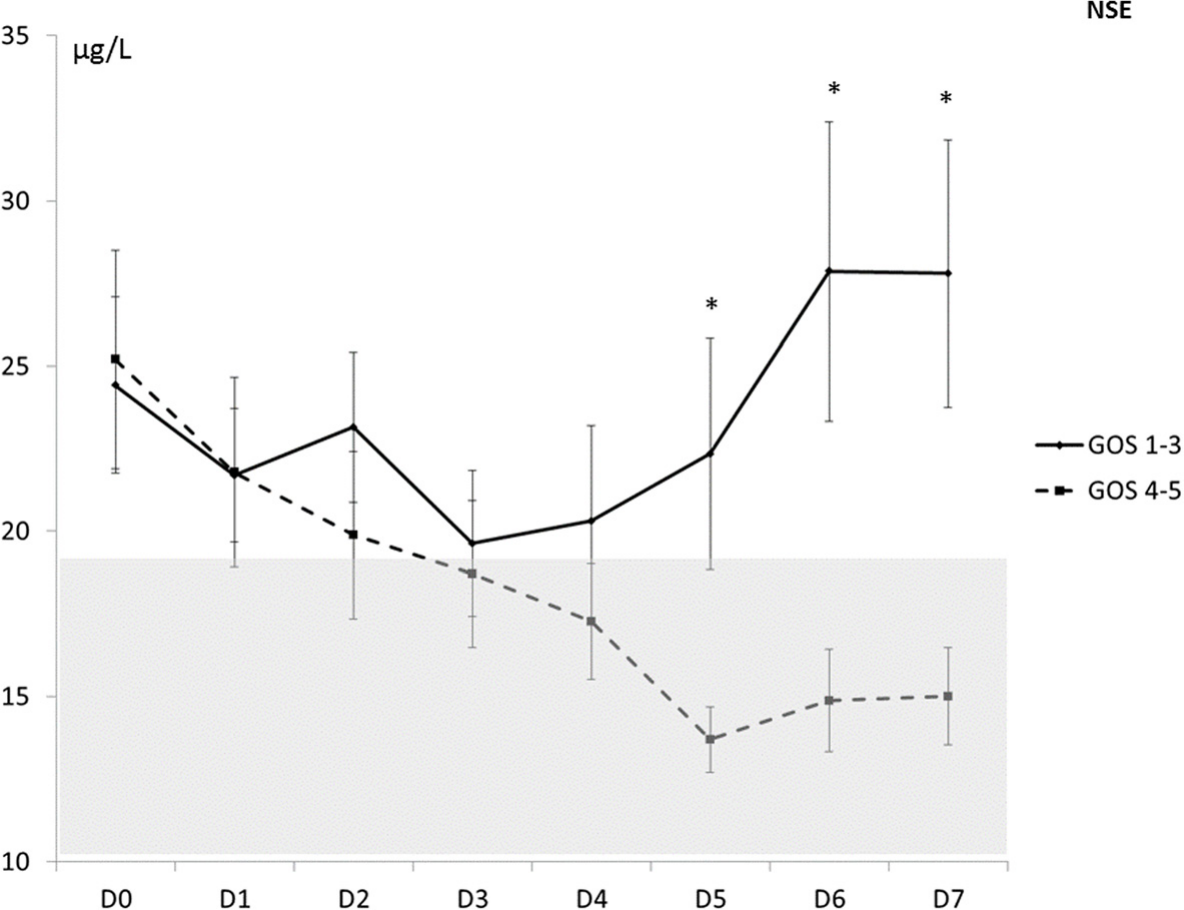
Time course values of neuron specific enolase NSE within the first week after subarachnoid hemorrhage in the groups with good or poor outcome (*p <0.05). *GOS* Glasgow outcome scale

The NSE value was significantly higher in the poor recovery than in the good recovery group from day 5 to day 7 (*p* <0.01). From baseline to day 5, there was no statistically significant difference between the two groups (Fig. 2).

We observed a significant association between the outcome group and the temporal course of S100 and NSE (respectively, *p* = 0.012 for PS100B and *p* = 0.011 for NSE).

### Threshold of PS100B and NSE for prognosis 6 months after SAH

**Fig. 3:**
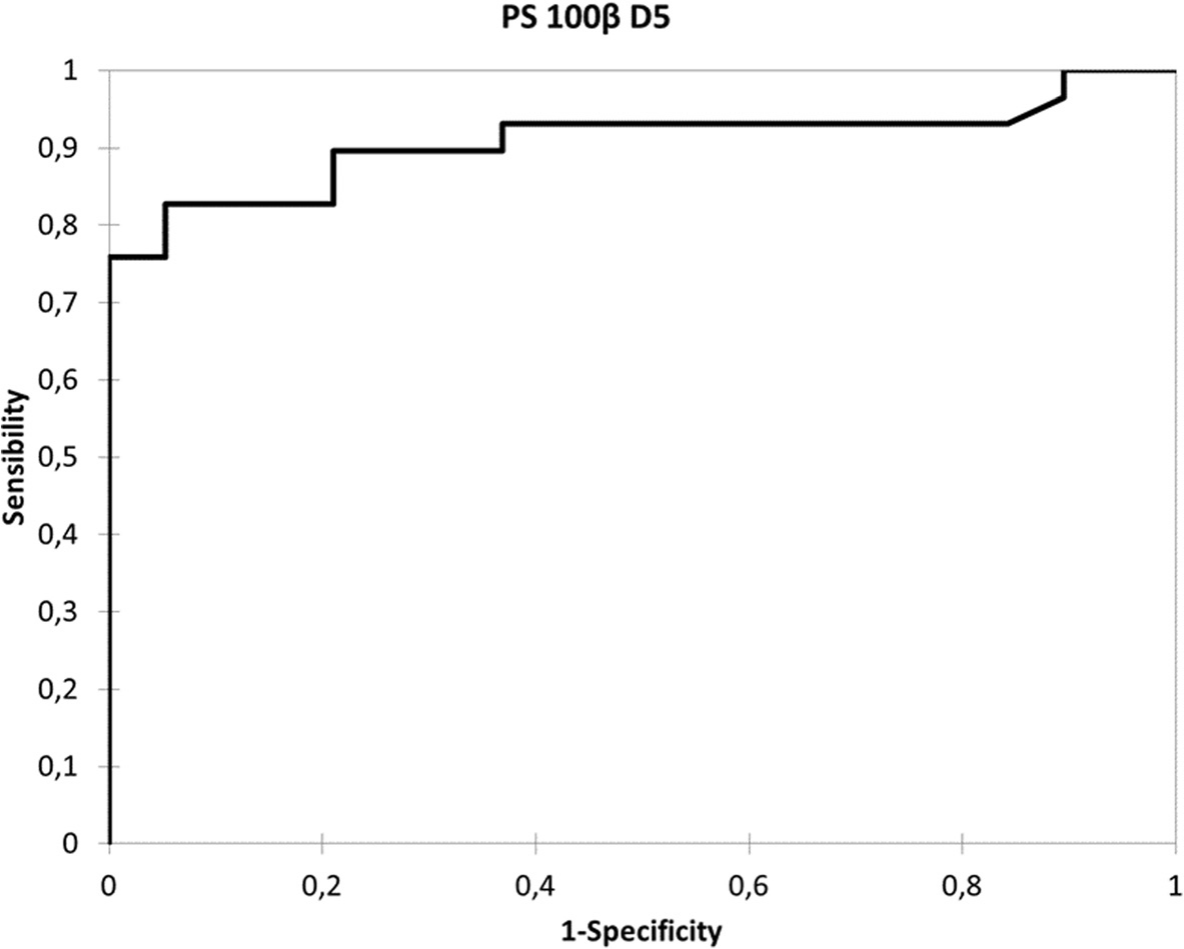
Receiver operating characteristic curve for 6-month prognosis by PS100β at day 5 (*D5*) after subarachnoid hemorrhage

Analysis of ROC curves showed that S100β at day 5 had the best area under the curve (AUC) for prediction of poor neurologic outcome 6 months after onset (Fig. 3). The AUC was 0.91 (0.83–0.99). For a threshold of 0.13 μg/L, sensitivity was 0.83 (0.65–0.93), specificity 0.95 (0.73–1.00), positive predictive value 0.96, negative predictive value 0.78 and false positive rate 2 %.

**Fig. 4:**
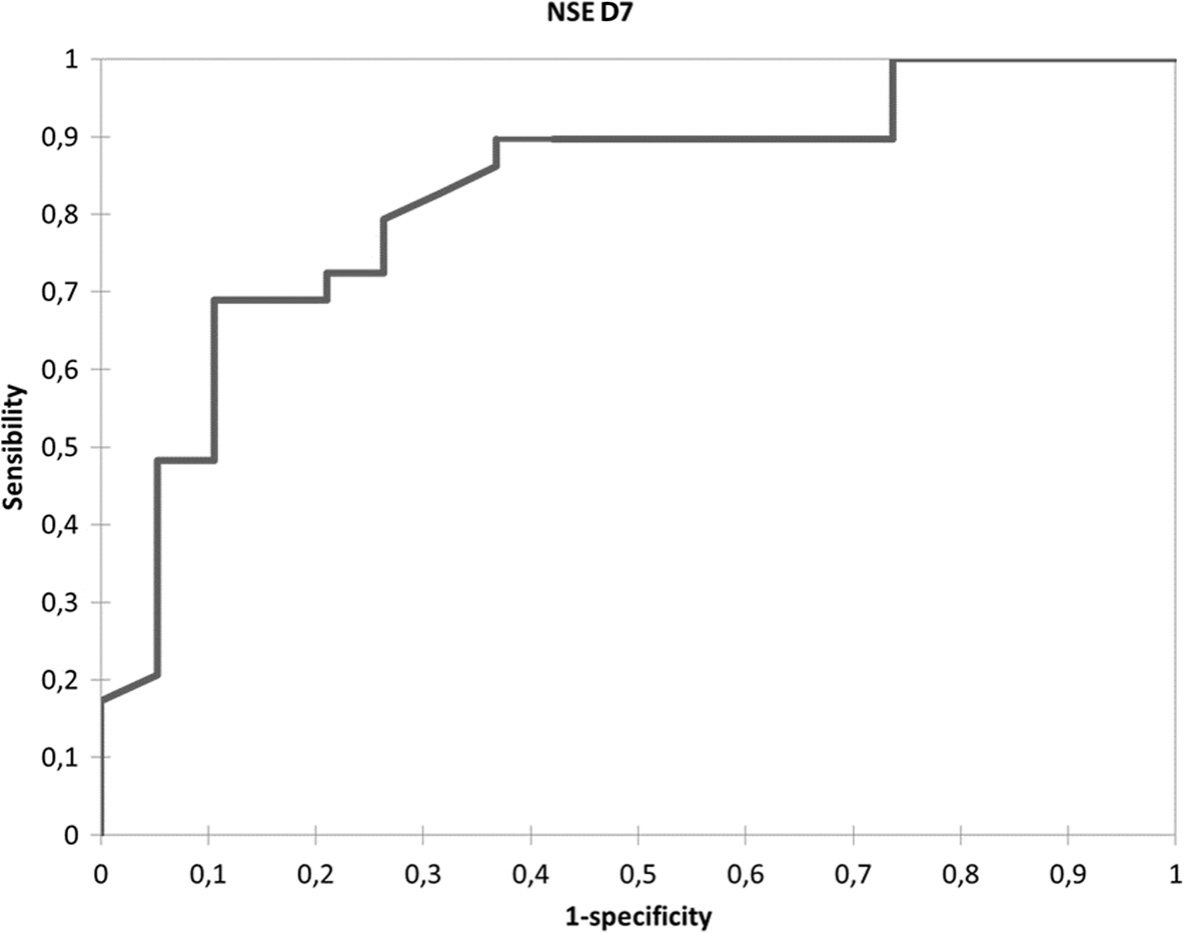
Receiver operating characteristic curve for 6-month prognosis by neuron specific enolase (*NSE*) at day 7 (*D7*) after subarachnoid hemorrhage

For NSE, the ROC curves showed at day 7 the AUC was 0.83 (0.71–0.94) (Fig. 4). For a threshold of 14.5 μg/L, sensitivity was 0.69 (0.51–0.83), specificity 0.89 (0.67–0.98), positive predictive value 0.9, negative predictive value 0.65 and false positive rate 4 %.

### Logistic regression analysis

On univariate analysis, four covariates - S100β at day 5, NSE at day 7, WFNS score and SAPS II - were identified as prognosis predictive factors 6 months after SAH (*p* <0,1). Although the sample size of patients admitted in our study was small, we entered all patients into logistic regression analysis. As shown in Table 2, only S100β at day 5 and SAPS II were significantly predictive of unfavorable outcome at 6 months, but not WFNS score or NSE at day 7. Association of independent parameters defined an AUC of 0.91, equivalent to S100β at day 5 alone.

**Table 2:** Multivariate analysis of risk factors for poor outcome at 6 months

Variables	Odds ratio (95 % confidence interval)	*p* value
SAPS II	0.934 (0.877–0.994)	0.03
PS100β at day 5	0.051 (0.003–1.029)	0.05
NSE at day 7	0.936 (0.855–1.025)	0.15
WFNS score	0.816 (0.093–7.18)	0.8

*SAPS* simplified acute physiology score, *NSE* neuron specific enolase, *WFNS* World Federation of Neurosurgical Societies

## Discussion

The major findings of this prospective study are summarized as follows: 1) both PS100β and NSE serum concentrations were higher in patients with poor prognosis than in those with good prognosis; 2) PS100β and NSE values did not normalize in patients with poor prognosis; and 3) a normal PS100β plasma concentration at day 5 after SAH and a low SAPS II were strongly and independently associated with a good neurological outcome at 6 months. To our knowledge, our study is the first to report serum concentration thresholds for two neurologic biomarkers, i.e., PS100β and NSE for prognostic assessment of patients presenting with SAH, admitted early after bleeding and treated by the endovascular technique.

### S100β and NSE: prognostic predictors of SAH

Identification of an early prognostic predictor is an important aspect in the management of patients with SAH. PS100β is a calcium binding protein that is present in the cytosol of astroglial and Schwan cells. This biomarker has been extensively studied in traumatic brain injury because of its good predictive value for long-term prognosis [8, 14, 20–24]. In a first study conducted by Wiessman et al. [25], this biomarker was found to be predictive of neurological prognosis in patients after SAH. With the evolution of treatment during the last 10 years and the development of endovascular techniques, Weiss et al. performed a study in 74 patients suffering from SAH [9]. The authors confirmed that the mean daily value of S100β assessed during the first 8 days post SAH, in association with clinical evaluation, can help the physician determine prognosis. This increased concentration was also reported for assessing the long-term outcome [15]. NSE has been less extensively studied in this context and results are more controversial. While increases in both S100β and NSE concentration have been observed in traumatic brain injury [26], Vos et al. did not identify NSE concentration at admission as a prognostic factor in patients with SAH [12]. These results were not confirmed in subsequent studies describing an association between NSE concentration and late prognosis, when NSE measurements were performed within several days after SAH [10]. In our study, we delibaretely selected patients with SAH admitted within the first 48 h after the first symptoms to reduce inter-individual variations in biomarker levels resulting from sample collected over a long period of time. We demonstrated that S100β quickly and significantly increased within 7 days after injury in patients with poor prognosis, confirming the important early predictive value of this biomarker. On the other hand, NSE concentrations were not statistically different between the two groups for the first 5 days post SAH, and we observed the first decrease in NSE within the first 3 days post SAH, in both groups of patients. Thereafter, from day 4 to day 7, we observed an increase in the NSE value only in the poor prognosis group. We could hypothesize that secondary insults might generate this phenomenon. We observed a significant increase in the number of patients with vasospasm in the poor prognosis group, occurring at day 5 [4–16, 19] (results not reported) and a rate of DCI in the poor prognosis group of 68 % compared to 10 % in the good prognosis group (*p* <0.01). As NSE and PS100 can be expressed in ischemic lesions, we may hypothesize that the higher rate and increase later after SAH could be explained in this way [5, 6]. However, it has been shown the increase in these biomarkers not only occurs after cerebral vasospasm or DCI during SAH, but sometimes in association with extracranial complications [19]. Only 6 patients among the 48 presented an acute respiratory detress syndrom as an extracranial complication. Therefore, we can expect that the variation in PS100 and NSE may be exclusively related to brain injury. These biomarkers could so be interesting predictors of new complications, such as vasospasm or DCI occurring in the course of SAH. Finally, due to the small number of patients, this hypothesis remains to be confirmed by further larger trials. Based on our results, such biomarker levels might be an additional tool to help the clinician advise the family on the patient’s prognosis and to make decisions about prolonged aggressive treatment or withdrawal of treatment. One could argue, however, that our results suggest that these biomarkers could predict outcome, but that they do not indicate whether they could create an opportunity to improve the outcome through adaptations in therapeutic measures. This study was not primarily designed to answer this question.

### Thresholds of S100β and NSE for prognosis

Previous studies proposed mean values of S100β during several days to assess the prognosis of these patients. However, this appears to be difficult to realize in clinical practice. The initial value has been described but the SAH is associated with secondary injuries during the days following the injury, that are not taken into account by the initial measurement alone. Definition of a threshold later in the course of SAH could thus be helpful for clinician decision-making.

### Association of predictors to improve prognosis

Association of different predictors have been proposed to improve the prognosis assessment. The WFNS clinical score demonstrated good accuracy for prognosis assessment [16]. In a recent study Turck et al. proposed a multi-parameter panel model including four brain injury-related proteins, one cardiac biomarker and the WFNS score. With this combination, the authors observed 70 % sensitivity and 90 % specificity when three of these parameters were abnormally high. Age has been also reported as a very interesting predictive marker of outcome in SAH patients [18]. In our study we found that both S100β measured at day 5 and SAPS II were good independent predictors of neurologic outcome, whereas WFNS was not. This could be explained by the high prevalence (60 %) of WFNS scores 1 and 2 in our population. The accuracy of PS100 β to determine the prognosis of patients with SAH was not improved by adding SAPS II.

### Limitations

Even though this study was the first to assess threshold values for SAH prognosis, there were several limitations. First, due to the small number of patients we report large inter-individual variations which could mean our study was underpowered, possbily influencing our results and conclusion. Thus, further large studies are needed to confirm these results. Moreover, these results cannot be considered for patients with SHA treated by surgical aneurysm clipping, or for those admitted more than 48 h after the subarachnoid bleeding. In our institution, coiling is systematically performed for SAH patients as the primary treatment and surgical clipping is intended as a second-line treatment or for special indications, such as anatomical considerations avoiding coiling, or intraparenchymatous hematoma requiring surgical treatment. During the study period no patients needed surgery. We performed only one measurement of each biomarker per day, which could be modified by systemic and laboratory variations. However, to limit this problem, each sample was compared with the previous sample and variation >20 % led us to discuss the opportunity to control the result on an additional sample.

We chose also to evaluate biomarkers within 7 days following SAH; even a longer period of monitoring could be interesting. However obtaining an early parameter to help the physician determine prognosis could be of interest and the thresholds at day 5 and 7 are thus clinically relevant. Whether a longer period, particularly for NSE, would lead to different results remains to be determined.

Moreover prognosis assessment was performed by phone call. This choice allowed us to have a 100 % follow up at 6 months, but limits the quality of the assessment because we chose to use GOS evaluation for its easy application by this method, even if other scales (GOSE, mRS) would be more accurate.

Others biomarkers have been studied in SAH. Troponin is also discussed in terms of prognosis in SAH. It has been proposed as an interesting predictive factor for SAH prognosis [27], but in other studies it has been described as having poor value as a prognostic marker [28]. We conducted an analysis of the initial cardiac biomarker, troponin Ic (results not reported). We did not observe a significant difference between patients with poor and good prognosis (1.1 ± 2.9 vs 1.8 ± 0.8 mmol/L; *p* = 0.3). However, we acknowledge the importance of the level of missing data.

In our study we did not assess serum glial fibrillary acidic protein (GFAP), an intermediate filament protein, specifically of astrocytic lineage, because of the availability of the assay, but it seems this biomarker could also be interesting to investigate. Indeed it has been described as a good prognosis marker at day 3 in patientS with SAH [29].

## Conclusions

To conclude, the need to have access to accurate biomarkers for prognosis assessment is essential to improve care in the critically ill patient. Indeed it could help physicians to adapt the level of therapy according to the predictive values of the biomarkers. S100β may be a reliable predictor of prognosis in SAH. We described a threshold at day 5 for assessment of neurological outcome. NSE could be another interesting predictor with a later prognostic value. However, in our study, combination of NSE or clinical predictors such as WFNS score or SAPS II, seems not to improve the independent predictive value of S100β.

## Key messages

- Both PS100β and NSE serum concentration were higher in patients with poor than with good prognosis
- PS100β and NSE values did not normalize in patients with poor prognosis
- A normal PS100β plasma concentration at day 5 following SAH and a low SAPS II were strongly and independently associated with a good neurological outcome at 6 months

## Additional file

Additional file 1 (DOC 64 kb)

## Abbreviations

AUC: area under the curve
CT: computed tomography
DCI: delayed cerebral ischemia
FeCO_2_: expiratory carbon dioxide
GCS: Glasgow coma scale
GFAP: glial fibrillary acidic protein
GOS: Glasgow outcome scale
ICP: intracranial pressure
IVH: intraventricular hemorrhage
kDa: kiloDalton(s)
NSE: neuron specific enolase
ROC: receiver operating characteristic
S100β: Protein S 100 β
SAH: subarachnoid hemorrhage
SAPS II: Simplified acute physiology score
SpO_2_: oxygen saturation
WFNS: World Federation of Neurosurgical Society
