# Supplementary material for: Early and persistent high level of PS 100β is associated with increased poor neurological outcome in patients with SAH: is there a PS 100β threshold for SAH prognosis?
Source: Crit Care. 2016 Feb 3;20:33. doi: 10.1186/s13054-016-1200-1 (PMC4738799; doi:10.1186/s13054-016-1200-1)
Supplement: Additional file 1 — (DOC 64 kb) [file 13054_2016_1200_MOESM1_ESM.doc]

**Additional file 1**

GOS used for phone assessment at 6 months


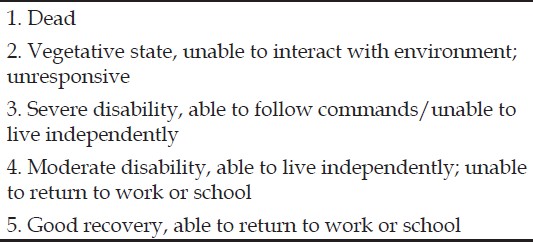


from Jennett B, Bond M. Assessement of outcome after severe brain damage. Lancet 1975;1:480-484
